# Exploring determinants of hand hygiene compliance in LTCFs: a qualitative study using Flottorps’ integrated checklist of determinants of practice

**DOI:** 10.1186/s13756-021-00882-2

**Published:** 2021-01-14

**Authors:** Dominique Lescure, Anja Haenen, Sabine de Greeff, Andreas Voss, Anita Huis, Marlies Hulscher

**Affiliations:** 1 Scientific Center for Quality of Healthcare, Radboud Institute for Health Sciences, Radboud University Medical Centre, P.O. Box 9101, 6500 HB Nijmegen, The Netherlands; 2 Centre for Infectious Disease Control/Epidemiology and Surveillance Unit, National Institute for Public Health and the Environment (RIVM), P.O. Box 9101, 6500 HB Nijmegen, Bilthoven, The Netherlands; 3 Department Antimicrobial Resistance and Healthcare Associated Infections, National Institute for Public Health and the Environment, P.O. Box 1, 3720 BA Bilthoven, The Netherlands; 4 Department of Medical Microbiology, Canisius-Wilhelmina Hospital, P.O. Box 9015, 6500 GS Nijmegen, The Netherlands; 5 Present Address: Erasmus MC, University Medical Center Rotterdam, PO Box 2040, 3000 CA Rotterdam, The Netherlands

**Keywords:** Focus groups, Hand hygiene, Long-term care facilities, Nursing staff, Qualitative research

## Abstract

**Background:**

Elderly residents in long-term care facilities (LTCFs) are vulnerable to healthcare-associated infections. Although hand hygiene is a leading measure for preventing infection, the compliance of healthcare workers is low. The aim of this study is to identify determinants that influence hand hygiene compliance of nursing staff in LTCFs. This information on determinants can eventually be used to develop a tailored implementation strategy for LTCFs.

**Methods:**

This is an explorative, descriptive study using qualitative methods. We performed semi-structured focus group discussions with 31 nurses and nurse assistants from five Dutch LTCFs. Our focus group discussions continued until no new information could be identified from the data. We used Flottorps’ comprehensive checklist for identifying determinants of practice (the TICD checklist) to guide data collection and analysis. The audiotapes were transcribed verbatim and two authors independently analysed the transcripts with Atlas.ti software.

**Results:**

LTCFs for the elderly have setting specific determinants that are decisive in explaining hand hygiene compliance. Most of these determinants are related to the residents with whom nurses build close relationships and for whom they want to create a homelike atmosphere. Residents can complicate the provision of care with unpredictable behaviour, being unwilling to receive care or use shared facilities. Our study also discovered setting-transcending determinants related to knowledge, professional interactions, guidelines, and incentives/resources.

**Conclusions:**

Nurses in LTCFs are constantly pursuing a balance between working hygienically, responding adequately to acute care needs, and maintaining a homelike environment for their residents. As a result, setting-specific determinants affect hand hygiene compliance, as do the known determinants that are important in other care settings. To improve compliance in LTCFs, interventions should be selected on a theoretical base while linking these determinants to change interventions.

***Trial registration*:**

Registration number 50-53000-98-113, Compliance With Hand Hygiene in Nursing Homes: Go for a Sustainable Effect (CHANGE) on ClinicalTrials.gov. Date of registration 28-6-2016.

## Background

Healthcare-associated infections (HAIs) represent a major threat to patient safety [1–3]. Especially in long-term care facilities (LTCFs), which focus on providing care for the elderly, residents are vulnerable to HAIs because they have weaker immune systems and live in close quarters [4–6]. Moreover, microorganisms can easily be transmitted because most residents use shared facilities, live in close proximity and often develop close relationships with other residents and the nursing staff. The European Centre for Disease Prevention and Control found that the crude prevalence of European residents with at least one HAI was 3.4% [7]. In 2016, the prevalence of Dutch residents living in LTCFs with one or more HAIs was 2.6% [8]. Hand hygiene is recommended as a pivotal measure for preventing HAIs [9]. Despite its importance, available data show that compliance of healthcare workers in LTCFs vary between 11 and 27% [10–12]. A French study, focusing on acute-care geriatric wards, skilled nursing facilities and LTCFs, found a higher rate of 61%, showing variation between settings [13]. It is fundamental to address the behavioural determinants that influence current compliance to effectively improve the hand hygiene compliance of healthcare workers [14, 15].

In daily practice, a wide range of determinants influence whether recommended care is provided or not. Flottorp et al. systematically synthesized current frameworks and taxonomies of factors that help or hinder improvements in healthcare [16]. These generic determinants can be grouped in seven domains: guideline factors; individual healthcare professional factors; patient factors; professional interactions; incentives/resources; capacity for organisational change; and social, political, and legal factors. Extensive research focusing on healthcare workers in hospitals and one study in paediatric LTCFs confirm that various determinants fitting in some of these domains, both barriers and facilitators, can indeed affect hand hygiene [17–24]. Recent quantitative studies show that individual healthcare professional determinants, such as beliefs about negative consequences and lack of knowledge, and the availability (or not) of incentives/resources (e.g. facilities and/or hand hygiene training) are also relevant in LTCFs for the elderly [25–28]. In addition, Hammerschmidt and Manser recently showed ‒in their mixed-methods study in German nursing homes‒ the influence of the availability of hand disinfectant materials in the immediate work area of nurses (i.e. incentives/resources) and of nursing managers having a role model function in daily practice (i.e. capacity for organisational change) [29].

Our study aimed to find out which determinants influence hand hygiene behaviour of nursing staff in Dutch LTCFs by comprehensively and systematically exploring all seven domains of Flottorp. We performed qualitative research as this helps to identify what range of determinants might influence hand hygiene behaviour of nursing staff in LTCFs for the elderly by establishing the reasons behind certain behaviours. This information on determinants can eventually be used to develop a tailored implementation strategy for LTCFs.

## Methods

This study is part of the CHANGE (Compliance with Hand hygiene in Nursing homes: Go for a sustainable Effect) project in 25 somatic, psycho-geriatric, and rehabilitation wards of 14 Dutch LTCFs [30]. These LTCFs are part of the national sentinel Surveillance Network for Infectious Diseases in Nursing Homes (SNIV) set up by the Dutch Centre for Infectious Disease Control of the National Institute for Public Health and the Environment RIVM [31]. The aim of CHANGE is to improve hand hygiene compliance of nursing staff with an improvement strategy that takes into account the behavioural determinants that explain current hand hygiene compliance. The focus groups were used to explore these determinants because they enable a broad examination of the hand hygiene context and stimulate dynamic interaction between participants. We used the consolidated criteria for reporting qualitative research checklist (COREQ) to organise, hold, and analyse the focus groups [32]. We took the wishes of each nursing team into account while choosing the time and place for the focus groups.

### Ethical approval

AHa composed a letter about the aim and content of the focus group, which the team managers sent to each participant. The participants were assured that participation was voluntary, and they could withdraw from the study at any time. Written informed consent was obtained from all participants at the focus groups. We removed references to individuals and LTCFs to anonymise the transcripts. The Regional Review Board for Human Research, Arnhem-Nijmegen, reviewed the CHANGE project and concluded ethical approval was not required under Dutch law (CMO no. 2015-2261).

### Focus group discussions

We used a combined purposeful-convenience sampling technique to recruit registered nurses and certified nurse assistants –via their team manager– from the LTCFs participating in the CHANGE project. Purposeful sampling is a technique widely used in qualitative research for the identification and selection of information-rich cases [33]. The team managers were requested to invite nurses with various opinions about hand hygiene to ensure that both positive and negative views would be represented and to give the nurses background information about the study in person. The team managers then gave the main researcher of the CHANGE project (AHa) contact details for the nurses who were willing to participate. AHa used this information to e-mail the nurses personal invitations. All the invited nurses agreed to participate. The focus groups started just before or after the work shift of the nursing team and lasted approximately 1.5 h at the LTCFs.

New LTCF wards were invited to participate in a focus group until data saturation (the point at which no new information or themes in the additional data were observed) was attained. The focus group moderator (AHa; PhD student, an infection prevention expert), was trained in qualitative research. There was no relationship established before the focus groups between the moderator and the participants. To begin the focus groups, the moderator introduced herself briefly as a public health researcher and explain the aim of the project. She did not mention her background as an infection prevention expert to avoid intimidation of the participants and to establish openness in talking about hand hygiene. To arrive at a common understanding about hand hygiene and to achieve our research aim we explained the World Health Organisation (WHO) hand hygiene definition for LTCFs before the start of each focus group [34].

The moderator used a semi-structured topic guide which centred on the main question: ‘What barriers do you encounter in maintaining hand hygiene in accordance with the national guideline?’ Flottorps’ comprehensive checklist distinguishing 57 determinants of practice grouped in seven domains (Table 1) served as inspiration for this guide [16].

**Table 1:** Flottorps’ checklist with domains of practice determinants

Domain	Subdomain	Definition
1. Guideline factors	Recommendation	E.g. clarity (clearness of the target population, the settings in which the recommendation is to be used, and the recommended action)^a^
2. Individual healthcare professional factors	Knowledge and skills	E.g. domain knowledge (the extent to which the targeted healthcare professionals have pre-existing knowledge or expertise about the targeted condition)^a^
3. Patient factors	Patient needs	Real or perceived needs and demands of the patient
4. Professional interactions	Communication and influence	The extent to which the targeted healthcare professionals’ compliance is influenced by professional opinions and communication
5. Incentives and resources	Availability of necessary resources	The extent of availability of resources required for compliance
6. Capacity for organisational change	Mandate, authority, accountability	The mandate, authority, and accountability for making necessary changes
7. Social, political, and legal factors	Economic constraints on the healthcare budget	Limits of the total healthcare budget or its growth

This table is based on Flottorp et al. [16]

^a^There are multiple subcategories for these subdomains. We show only one example in the table

The participants did not receive questions before the focus group and were not informed about the use of Flottorps’ checklist for analysing the data. Furthermore, the participants received no reimbursement for participating.

### Data analysis

Analysis was ongoing throughout the study: shortly after each focus group, the moderator summarised the interpretative and analytical notes for evaluating and, when necessary, refining the topic guide with help from an expert in qualitative research. Thus, we ensured that each domain of Flottorps’ checklist was discussed in the focus groups. An independent transcription company transcribed the focus groups audiotapes verbatim. To enhance the reliability and validity, AHa and DL (Ph.D. student, experience with qualitative research), used Atlas.ti software to independently analyse the transcripts. We categorised the assigned codes in the seven main themes of Flottorps’ checklist using an approach of thematic content analysis. Existing discrepancies were discussed until consensus was reached. A third author, AHu (senior researcher, experienced in qualitative research), was consulted to reach consensus in unresolved discussions.

## Results

Five focus groups were held between 30 May and 21 September 2016 with five or six participants per group (Table 2).

**Table 2:** Characteristics of focus group participants

	Focus group participants (N = 31)
Gender	
Women	30 (97%)
Man	1 (3%)
Age	
< 25 years	2 (6%)
26–35	9 (30%)
36–45	6 (20%)
46–55	9 (30%)
> 56 years	5 (16%)
Education	
Registered nurse	25 (81%)
Certified nurse assistant	6 (19%)
Years of experience	
< 10 years	10 (33%)
11–20	10 (33%)
21–30	7 (23%)
> 30 years	4 (13%)
Special position	
Hygiene quality officer	7 (23%)

The moderator’s interpretative and analytical notes after the first focus group showed that, in describing their hand hygiene compliance, participants automatically discussed using gloves in specific situations and did not consider washing or disinfecting hands or the different moments of hand hygiene. After explaining the WHO definition of hand hygiene in the following focus groups, the participants discussed all elements of hand hygiene.

The determinants discussed in the focus groups were clustered in the first six main themes of Flottorps’ checklist [16]. We did not find determinants that were related to the seventh theme: social, political, and legal factors (Table 3).

**Table 3:** Quotes related to the main themes of Flottorps’ comprehensive checklist

Flottorps’ main themes	Subthemes	Category	Representative quotes
1. Guideline factors	Recommendation	Clarity	‘Guidelines are fine if they are concise and clear. But not when I have to read a whole letter-sized page before I can begin. I would like to have a picture with five bullet points instead of the three pages we have now.’ (focus group 5)
		Accessibility of the recommendation	‘There is a protocol for hand hygiene.... First you have to start your computer to search for the protocol. It has not been posted in a visible spot.’ (focus group 5)
2. Individual health professional factors	Knowledge and skills	Domain knowledge	‘I suppose that alcohol-based handrub is good for after washing your hands. But if you do not wash hands and only use the handrub, that is not good.’ (focus group 1)
	Cognitions (including attitudes)	Expected outcome	‘Yes, I believe that, but it [hand hygiene] will not prevent 100% of everything [infections]. I do not believe that.’ (focus group 2)
		Intention and motivation	‘I put on gloves to protect myself. I have not really thought about it [hand hygiene] being necessary for the resident, I do it purely for myself. More for myself than for the resident.’ (focus group 4)
		Emotions	‘I am very sensitive to someone’s hygiene or lack of it. This morning I realised that I had washed my hands four times while I treated that man.’ Interviewer: ‘Can you describe the poor hygiene of a resident?’ ‘Some residents cannot take care of themselves or are too passive or inactive, and I know the nurses do that for them. I take other things into account: what their nails look like and how they smell.... It is very subjective, of course.’ (focus group 2)
	Professional behaviour	Nature of the behaviour	‘Then you are busy with the resident, and sometimes you think, oh, I have to go get that. Then you have to take off [the gloves], wash your hands, go outside.... It should be done, but it is not done.’ (focus group 3)
3. Patient factors	Patient needs		‘Definitely with us on the psycho-geriatric ward [there is a lot of physical contact with residents]. There is a lot of cuddling.’ (focus group 2)
	Patient preferences		‘Yes, but I believe that when I shower someone with gloves on, it creates a distance.... Then I think how would I like it, and I would not like it if someone approached me with those blue gloves on.’ (focus group 1)
	Patient behaviour		‘I believe that washing my hands beforehand is not relevant at the moment [when a resident suddenly becomes unwell].... It so happened that I was outside to eat and smoke, and when I came back in a woman became unwell.... My hands were not clean, but the woman came first.’ (focus group 4)
4. Professional interactions	Communication and influence		‘Look, if you see that your colleague is not washing her hands, after, for instance, faeces, you think: What a filthy colleague... but you do not always see that. If someone is working alone, then I cannot check whether she has washed her hands.’ (focus group 4)
5. Incentives and resources	Availability of necessary resources		‘Anyway, it is encouraging that soap and towels are available in each resident’s room. That is encouraging, but they need to be replenished. That does not always happen.’ (focus group 1)
	Continuing education system		Interviewer: ‘Did you receive any education about hand hygiene or did the geriatrics specialist provide information?’ ‘I don’t know.’ (focus group 1, Participant 1) ‘No, he only said to me one time, “You have to wash your hands or else it is not effective”’. (focus group 1, Participant 2)
6. Capacity for organisational change	Regulations, rules, policies		‘The cleaning staff used to do a lot, cleaning the kitchens, etc. Economising has done away with all that. So they have to do the same work in less and less time. A lot of things have now been assigned to the care that we have to do ourselves.’ (focus group 5)
	Priority of necessary change		‘Alcohol [handrub] was only available during the Noro virus outbreak. Everything is ready for something like that. We are only really alert when there is an outbreak.’ (focus group 1)
	Monitoring and feedback		‘It’s a pity that there is no feedback. But if we had known that we had really bad marks, maybe we would...’ (focus group 4, Participant 1) ‘Have given it more attention.’ (focus group 4, Participant 2)

Adapted from Flottorps’ et al. [16] checklist

### Guideline factors

#### Recommendation

The participating nurses discussed the availability, accessibility, and content of the hand hygiene guidelines. In one focus group it was mentioned that the guidelines could only be accessed online, which was often impractical. The nurses in other focus groups stated that their organisations’ guidelines could easily be obtained. However, almost all nurses admitted they never consulted guidelines because they are long-winded and/or too complicated. They suggested that guidelines should contain more illustrative explanations and should use simple wording. Some nurses described instruction posters for bringing guidelines to their attention. Not all nurses agreed; some questioned the efficiency of such reminders.

Then they thought up placing a note at each sink with a germ cartoon saying ‘Wash your hands!’ They wanted to replace the cartoon from time to time, but this is outdated and doesn’t work. (focus group 5)

### Individual healthcare professional factors

#### Knowledge and skills

Except nurses responsible for infection prevention in their wards (hygiene quality officers), most nurses were uncertain about when and how to clean their hands. One common misconception was that using soap and water was obligatory and could not be replaced with alcohol-based handrub. Some nurses were reluctant to wash their hands frequently because of possible skin irritations or wounds this could cause. Many nurses mistakenly believed that gloves were a substitute for hand washing or disinfection.

I consciously put on my gloves for everybody, and I take them off afterwards, but I do not wash my hands. I have to admit that, now that I have thought of it. (focus group 3)

#### Cognitions (including attitudes)

Nurses’ cognitions were a recurrent focus group theme. Most nurses were convinced that proper hand washing reduced the risk of transmitting infections. They also believed, however, that hand washing alone was insufficient.

Is it worth the effort, all that hand washing, indeed. If the rest [materials] is not cleaned and you only manage that bit of hand washing? How efficient is that? (focus group 5)

None of the nurses were aware of scientific evidence for the causal relation between hand hygiene and infections, and some were even unsure whether their own compliance was related to the occurrence of infections.

Sometimes I think: I am not sick, so it will be ok. And the residents are all alive. Good for their immune system. (focus group 1).

The nurses underlined the need to better understand the effectiveness of hand hygiene by making the presence of micro-organisms visible with ultraviolet lamps.

An important influence on hand hygiene was most nurses’ intention to protect themselves against HAIs. Some explicitly stated they were more concerned with protecting their own health and that of their colleagues than preventing residents.

Nurses emotions also affect their hand hygiene compliance. This theme was thoroughly discussed in all focus groups. Nurses stated that they prefer to clean their hands in areas inaccessible to residents. They felt that materials could be contaminated by micro-organisms when touched by residents. Moreover, most nurses said they were more inclined to wash or disinfect their hands when they perceived a specific care task or resident as ‘dirty’.

In the night shift I remove the stockings of all residents and get them changed. One resident has excessively sweaty feet, so then I wash my hands, but after changing the other residents’ clothes I do not. (focus group 1).

#### Professional behaviour

Not all nurses planned their work activities in advance. A few admitted that this made them forget to collect all the necessary materials before they began looking after the residents. This led to impractical situations—they had to collect forgotten materials in a storage room. Then, after touching multiple surfaces, they forgot to wash their hands again before starting their care tasks.

A few nurses admitted that they sometimes simply forgot hand hygiene.

### Patient factors

#### Patient needs

The focus groups showed that nurses developed intimate and caring relationships with residents. All the nurses noted the common acceptance of being close with residents and cuddling them once in a while. Some nurses explained that these social contacts discouraged them from wearing gloves or frequently washing their hands.

I do not hesitate to cuddle residents. You know that I love cuddling residents. (focus group 1)

#### Patient preferences

Generally, nurses felt that compliance with hand hygiene guidelines upsets residents and creates a distance between them. However, a few nurses who were asked about residents’ reactions to their hand hygiene compliance said that residents did not complain. While two nurses described the residents’ willingness to remind them of hand hygiene, the other nurses assumed that most residents were unaware of the necessity of wearing gloves or disinfecting hands. A few nurses from a somatic ward suggested that residents should be better instructed about hand hygiene so they could remind staff members to disinfect their hands.

#### Patient behaviour

Several nurses explained how unpredictable resident behaviour of residents complicated their compliance to hand hygiene guidelines.

The time I didn’t wash my hands was when I emptied the catheter bag and another resident stood up. Then I needed to help him because he was at high risk of falling. And afterwards, another resident called, and then I forgot. (focus group 4)

Most nurses identified the residents’ emotional, physical, and mental demands as challenges and deterrents to hand hygiene. Some nurses reported that some residents tried to escape or refuse care. Consequently, nurses depended on moments when these residents were willing to receive care and might have omitted proper hand hygiene because they had to act quickly.

That was a resident with whom you really had to grab the moment. Then you don’t think: “First I have to wash my hands, put on my gloves.... (focus group 4)

Another complicating factor was that residents could wander freely throughout the LTCF and use shared facilities at will. Nurses feel these residents might transmit micro-organisms to each other and to the facilities. One nurse explained that there was no guarantee that residents would comply when she encouraged them to wash their hands after a toilet visit.

Moreover, some nurses in the psycho-geriatric wards emphasised the fact that hand hygiene materials were difficult to obtain or were locked away to prevent mentally disturbed residents from hurting themselves (e.g. by drinking soap or hand alcohol that looks like water). This hindered the performance of hand hygiene.

### Professional interactions

#### Communication and influence

Most nurses usually worked alone in shifts. This made them unaware of their colleagues’ hand hygiene habits and hindered their addressing each other’s compliance. However, if they noticed a colleague’s incorrect hand hygiene, they all would point it out. They agreed that direct feedback about peers’ compliance might be a good incentive for better hand hygiene. Nurses who had already criticised colleagues said they did so indirectly or as a joke to make it less embarrassing. All nurses acknowledged that it is crucial to create an open atmosphere of communication with room for receiving feedback.

Some nurses said they felt uncomfortable criticising when collaborating with superiors such as a physician or physiotherapist. Some thought other professionals’ hand hygiene was their own responsibility.

Some nurses observed that other professionals (at the same or a higher level) neglected hand hygiene at recommended times but indicated that this had no negative influence on their own hand hygiene.

### Incentives and resources

#### Availability of necessary resources

The nurses unanimously agreed that the availability of hand hygiene materials was an essential precondition for compliance. Overall, they concluded that tissues, soap, and handrub were adequately available in each resident room. However, these materials were not properly replenished.

If I am on the ward, I have to open two doors to find one [alcohol-based handrub]. If it has been refilled. They are often empty. (focus group 5).

Nurses were concerned about the insufficient supply of gloves and hand alcohol in their LTCFs. Moreover, in LTCFs where these materials were present, but not in strategic locations, their use was hindered.

Another barrier is that there aren’t sinks everywhere; for instance, not in the hallway. (focus group 3).

The focus groups showed that this applies particularly to LTCFs where the management pursues a homelike setting. Some nurses criticised this policy because, consequently, materials were not provided everywhere. Sinks and alcohol-based handrub should be conveniently located near residents’ rooms for easy use. Another solution, which one focus group praised, was aprons with which nurses could carry small personal containers of alcohol-based handrub with them.

They all preferred alcohol-based handrub, which causes less skin irritation. One group complained that these handrubs were exclusively provided in personal containers only during outbreaks. Opinions were divided as to whether this was indeed the policy. A few nurses suggested that wearing personal containers was unsterile because you use your contaminated hands to open them.

In one focus group it was stated that available cleaning materials were not always accessible and only available in specific ‘cleaning’ rooms, such as laundry rooms, where they were often blocked by other equipment and furniture.

No, there are carts there. Then I walk back with my laundry basket, and I put it down, and then wash my hands. (focus group 2)

The focus groups also discussed human resources; understaffing was an important underlying cause of omitting hand hygiene. There were too few staff members to give each resident adequate care and attention, especially when they were responsible for multiple residents. Busy shifts were an important cause of forgetting hand hygiene at crucial moments. It was not really a matter of forgetfulness, but of prioritising acute care for vulnerable residents above hand hygiene.

The last resource discussed in all focus groups, time, was crucial to hand hygiene. It was not always feasible for nurses to comply with the guidelines due to the substantial amount of time they had to invest in hand hygiene and the limited time available for all care activities.

One barrier may be that it takes quite a long time to be sure that you cleaned [your hands] well. Someone said 10 s, but I also heard 30 s. Well, if you keep track of the time and you wash your hands for 30 s, that won’t happen. That’s too long. (focus group 3).

Because of the efforts required to comply with the guidelines, some nurses consciously chose to omit hand hygiene.

#### Continuing education system

Some nurses had not received enough education about appropriate hand hygiene. They claimed this could be solved with continuous education in the LTCF, on condition that it was obligatory for all nursing staff. Another way to raise hand hygiene awareness would be an introductory course for new employees. In contrast, there were nurses from one focus group who received extensive hand hygiene training and had to pass an exam on their hand hygiene performance.

### Capacity for organisational change

#### Regulations, rules, policies

Overall, budgetary economising caused problems. There was an inadequate budget for working staff and the purchase of necessary equipment, which led to more work pressure and less hand hygiene compliance.

One focus group reported they suspected solving employee or facility-related problems had become difficult because of the switch to self-directed teams. Nurses in these teams have less authority and fewer tools to make changes in the organisation.

Until now, we have always worked with team leaders. So, if you run into problems, you ask your colleagues: “Do you have the same problems?” Well, then we have a look to see whether money is available for buying material or equipment. Often there is no money, so there you are. (focus group 3)

However, most were optimistic because of a coach assigned to help them with various problems. Two other nursing teams in a similar transition confirmed that they foresaw no negative effects on hand hygiene from working in self-directed teams.

#### Priority of necessary change

The nurses needed frequent attention to hand hygiene spread throughout the year. Most said that giving hand hygiene more attention on paper (posters or flyers) would only have a detrimental effect, so the focus groups suggested various other methods: more attention to hand hygiene in team meetings, e-learning, and open observations. Furthermore, hand hygiene should not be reserved for outbreaks; it should be a main focus point in daily practice. In one LTCF, hand hygiene was already commonly discussed thanks to the infection-prevention expert or the hygiene quality officer.

#### Monitoring and feedback

One or more nurses in each focus group stated it was uncommon for their ward leader to provide them with feedback about infection rates in their ward or to discuss hand hygiene.

Last year there was a survey about hand hygiene compliance. It’s just a pity that we never heard anything more about it. If we had known that we got really low marks, we could have given it some extra attention. (focus group 4)

All the nurses assumed that feedback would motivate them to be more aware of their hand hygiene and the consequences of poor hand hygiene.

## Discussion

Our study aimed to find out which determinants influence hand hygiene behaviour of nursing staff in Dutch LTCFs. It showed that nurses in LTCFs are constantly pursuing a balance between working hygienically, responding adequately to acute care needs, and maintaining a homelike environment for their residents. Consequently, many determinants affect their hand hygiene compliance. This makes compliance with guidelines challenging. The determinants we found were related to all the main domains of Flottorp’s checklist except the domain ‘social, political and legal factors’ [16].

Knowing LTCF setting specific determinants is important to achieve hand hygiene compliance. Unlike hospitals, LTCFs are established to provide long-term care for their residents. Therefore, nurses in LTCFs must combine providing care with maintaining a homelike environment in which the needs of individual residents are met. Care moments must be tailored to the residents’ wishes and behaviour. Moreover, elderly residents in LTCFs have complex care needs, and especially those with psycho-geriatric disorders influence the planning and organisation of care. For example, hiding cleaning products for the residents’ safety impedes nurses’ hand hygiene compliance.

Another complicating LTCF-related determinant is the nurses’ working situation. Whereas nursing staff in hospitals often work in teams and are influenced by the behaviour of colleagues, nurses in LTCFs often work alone and comply according to their own norms, uninfluenced by team interactions [17, 35]. The recent Dutch switch to self-directed teams could unintentionally complicate team interactions and hand hygiene compliance. Although most nurses in our study had no major concerns regarding this switch, self-directed teams can become an impediment. A hierarchical leader is sometimes crucial for overcoming various barriers, such as disagreements between colleagues or conflicting priorities within the organisation. A leader could also ensure frequent attention to hand hygiene compliance, which –according to our participants– is currently lacking.

In addition to the above mentioned LTCF-related determinants, we have found that nurses’ cognitions are decisive in hand hygiene compliance. Emotions are crucial because they intertwine with all the decisions nurses make. In addition to personal beliefs about appropriate hand hygiene methods and ‘dirty’ tasks, perceptions of which rooms are suitable for hand hygiene and which residents require better hand hygiene influence nurses. Moreover, most nurses felt powerless. They were willing to change, but they felt their hand hygiene compliance could not improve unless other factors in the LTCF ward changed simultaneously. Residents undid the nurses’ hygienic efforts with contaminated hands, and managers provided insufficient attention to hand hygiene practices. Regarding the latter, Hammer Schmidt et al. also described how compliance depended upon role modelling from nursing managers [29]. Despite a general awareness of the impact of leadership on staff behaviour, nursing managers struggled with taking this role.

We also identified some setting-transcending barriers in concordance with previous studies focusing on hand hygiene compliance in LTCFs for the elderly. We identified determinants –also present in hospitals—that were related to guidelines, availability of materials and incentives/recourses, for example a scarcity of continuous hand hygiene training [18, 19, 26–29]. Similarly, determinants such as forgetfulness, lack of time, considering gloves as a substitute for good hand hygiene, and lack of hand hygiene materials are also present in hospitals, paediatric LTCFs, and LTCFs for the elderly [18, 19, 21, 29].

This study has several limitations that must be taken into account while interpreting our results. Although purposeful sampling is a technique widely used in qualitative research for the identification and selection of information-rich cases, we cannot rule out the possibility that team managers influenced the FGD participants [33]. However, as neither the team manager nor the participants were informed in advance of the exact topics of the discussion, and we obtained a wide range of both facilitators and barriers in most domains of the Flottorp checklist. We believe that, overall, we included participants with various opinions about hand hygiene.

Another limitation of our study is that only one man participated in the focus groups. Although we obtained a wide range of determinants in most domains of the Flottorp checklist, we may have missed some barriers or facilitators because of this imbalance.

Finally, the focus groups were restricted to LTCFs that participate voluntarily in the SNIV. The extra attention these facilities pay to preventing HAIs might have resulted in better informed and educated nursing staff, leading to an underestimation of determinants.

Our study may have implications for future interventions. Understanding the determinants of hand hygiene performance is a prerequisite for designing multimodal hand hygiene improvement strategies-like the WHO hand hygiene multimodel improvement strategies-that take these determinants into account. Taking the various determinants regarding *capacity for organisational change* as an example, would suggest the following combination of improvement strategies: managers should be trained to set clear norms, standards and goals which are regularly promoted taking into account the influence of residents on hand hygiene. Managers should also establish a monitoring and feedback system, and nurses need to be empowered to speak up when non-adherence is observed and should be actively involved in improving hand hygiene by playing an exemplary role.

We also suggest to consider glove use in tandem with hand hygiene in future interventions. In recent years, there has been a successful campaign in the Netherlands to wear gloves. However, the downside is-as also shown in our study-that workers feel protected and do not change their gloves on time or do not apply hand hygiene when taking them off. Loveday et al. have shown in hospitals that there is an increasing risk of cross-contamination by these inappropriate behaviours [36].

## Conclusion

The specific LTCF setting, where nurses pursue a homelike environment while providing care to vulnerable and mobile residents who can behave unpredictably, leads to a complex variety of determinants that influence nurses’ hand hygiene compliance. Not only do individual health-professional factors and resident-related factors contribute greatly, professional interactions, guidelines, organisation of care, and incentives and resources are also important. Our qualitative method, using Flottorps’ checklist, led to in-depth knowledge of the determinants that influence hand hygiene compliance. This information is invaluable for developing an improvement strategy for LTCFs that simultaneously should focus on the nursing staff, the organisational environment in which they work, and the people they work with. The improvement strategy will preferably consist of a multi-model approach, like the customized to the LTCF setting.

## Data Availability

The datasets used and analysed during the current study are available from the corresponding author on reasonable request.

## Abbreviations

LTCF: Long-Term Care Facilities
CHANGE: Compliance with Hand Hygiene in Nursing Homes: Go for a Sustainable Effect
HAI: Healthcare-associated infections
SNIV: Surveillance Network for Infectious Diseases in Nursing Homes
RIVM: National Institute for Public Health and the Environment
COREQ: Consolidated criteria for reporting qualitative research checklist
WHO: World Health Organisation
ZonMW: The Netherlands Organisation for Health Research and Development

## References

[1] Report on the Burden of endemic health care-associated infection worldwide: clean care is safer care. World Health Organization. 2011.

[2] Allegranzi B, Nejad SB, Combescure C, Graafmans W, Attar H, Donaldson L (2011). Burden of endemic health care-associated infection in developing countries: systematic review and meta-analysis. The Lancet.

[3] Burke JP (2003). Infection control—a problem for patient safety. N Engl J Med.

[4] Castle SC (2000). Clinical relevance of age-related immune dysfunction. Clin Infect Dis.

[5] Strausbaugh LJ (2001). Emerging health care-associated infections in the geriatric population. Emerg Infect Dis.

[6] Gavazzi G, Krause KH (2002). Ageing and infection. Lancet Infect Dis.

[7] Point prevalence survey of healthcare-associated infections and antimicrobial use in European long-term care facilities. European Centre for Disease Prevention and Control; 2014. 10.2807/1560-7917.ES.2018.23.46.1800394 PMC6247460 30458913

[8] Eikelenboom-Boskamp A, Saris K, Loosbroek MV, Drabbe MIJ, Jongh FD, Jong JWDD (2019). Prevalence of healthcare-associated infections in Dutch nursing homes: follow-up 2010–2017. J Hosp Infect.

[9] WHO guidelines on hand hygiene in health care: first global patient safety challenge clean care is safer care. World Health Organization; 2009. 23805438

[10] Ho ML, Seto WH, Wong LC, Wong TY (2012). Effectiveness of multifaceted hand hygiene interventions in long-term care facilities in Hong Kong: a cluster-randomized controlled trial. Infect Control Hosp Epidemiol.

[11] Liu WI, Liang SY, Wu SF, Chuang YH (2014). Hand hygiene compliance among the nursing staff in freestanding nursing homes in Taiwan: a preliminary study. Int J Nurs Pract.

[12] Smith A, Carusone SC, Loeb M (2008). Hand hygiene practices of health care workers in long-term care facilities. Am J Infect Control.

[13] Eveillard M, Raymond F, Guilloteau V, Pradelle MT, Kempf M, Zilli-Dewaele M (2011). Impact of a multi-faceted training intervention on the improvement of hand hygiene and gloving practices in four healthcare settings including nursing homes, acute-care geriatric wards and physical rehabilitation units. J Clin Nurs.

[14] Grimshaw JM, Eccles MP, Lavis JN, Hill SJ, Squires JE (2012). Knowledge translation of research findings. Implement Sci.

[15] Grol R, Wensing M, Eccles M, Davis D (2013). Improving patient care: the implementation of change in health care.

[16] Flottorp SA, Oxman AD, Krause J, Musila NR, Wensing M, Godycki-Cwirko M (2013). A checklist for identifying determinants of practice: a systematic review and synthesis of frameworks and taxonomies of factors that prevent or enable improvements in healthcare professional practice. Implement Sci.

[17] Erasmus V, Daha TJ, Brug H, Richardus JH, Behrendt MD, Vos MC (2010). Systematic review of studies on compliance with hand hygiene guidelines in hospital care. Infect Control Hosp Epidemiol.

[18] Erasmus V, Brouwers W, Beeck EF, Oenema A, Daha TJ, Richardus JH (2009). A qualitative exploration of reasons for poor hand hygiene among hospital workers: lack of positive role models and of convincing evidence that hand hygiene prevents cross-infection. Infect Control Hosp Epidemiol.

[19] Huis A, Holleman G, Achterberg T, Grol R, Schoonhoven L, Hulscher M (2013). Explaining the effects of two different strategies for promoting hand hygiene in hospital nurses: a process evaluation alongside a cluster randomised controlled trial. Implement Sci.

[20] Lankford MG, Zembower TR, Trick WE, Hacek DM, Noskin GA, Peterson LR (2003). Influence of role models and hospital design on hand hygiene of health care workers. Emerg Infect Dis.

[21] Loyland B, Wilmont S, Hessels AJ, Larson E (2016). Staff knowledge, awareness, perceptions, and beliefs about infection prevention in pediatric long-term care facilities. Nurs Res.

[22] Pittet D, Simon A, Hugonnet S, Pessoa-Silva CL, Sauvan V, Perneger TV (2004). Hand hygiene among physicians: performance, beliefs, and perceptions. Ann Intern Med.

[23] Wandel DD, Maes L, Labeau S, Vereecken C, Blot S (2010). Behavioral determinants of hand hygiene compliance in intensive care units. Am J Crit Care.

[24] Whitby M, McLaws M, Ross MW (2006). Why healthcare workers don’t wash their hands: a behavioral explanation. Infect Control Hosp Epidemiol.

[25] Aiello AE, Malinis M, Knapp JK, Mody L (2009). The influence of knowledge, perceptions, and beliefs, on hand hygiene practices in nursing homes. Am J Infect Control.

[26] Ashraf MS, Hussain SW, Agarwal N, Ashraf S, El-Kass G, Hussain R (2010). Hand hygiene in long-term care facilities: a multicenter study of knowledge, attitudes, practices and barriers. Infect Control Hosp Epidemiol.

[27] Castle N, Handler S, Wagner L (2013). Hand hygiene practices reported by nurse aides in nursing homes. J Appl Gerontol.

[28] Smith JD, Corace KM, MacDonald TK, Fabrigar LR, Saedi A, Chaplin A (2018). Application of the theoretical domains framework to identify factors that influence hand hygiene compliance in long-term care. J Hosp Infect.

[29] Hammerschmidt J, Manser T (2019). Nurses’ knowledge, behaviour and compliance cobcerning hand hygiene in nursing homes: a cross-sectional mixed-methods study. BMC Health Serv Res.

[30] Compliance with HAnd hygiene in Nursing homes: Go for a sustainable Effect (CHANGE). The Netherlands Organization for Health Research and Development (ZonMw); 2014.

[31] Haenen A, Verhoef LP, Beckers A, Gijsbers EF, Alblas J, Huis A (2019). Surveillance of infections in Long-Term Care facilities (LTCFs): the impact of participation during multiple years on healthcare associated infection incidence. Epidemiol Infect.

[32] Tong A, Sainsburry P, Craig J (2007). Consolidated criteria for reporting qualitative research (COREQ): a 32-item checklist for interviews and focus groups. Int J Qual Health Care.

[33] Palinkas LA (2015). Purposeful sapling for qualiiative data collection and analysis in mixed method implementation research. Adm Policy Ment Health.

[34] Hand hygiene in outpatient and home-based care and long-term care facilities: a guide to the application of the WHO multimodal hand hygiene improvement strategy and the ‘My Five Moments for Hand Hygiene’ approach. World Health Organization; 2012.

[35] Sax H, Uckay I, Richet H, Allegranzi B, Pittet D (2007). Determinants of good adherence to hand hygiene among healthcare workers who have extensive exposure to hand hygiene campaigns. Infect Control Hosp Epidemiol.

[36] Loveday HP, Singleton SLJ, Wilson J (2014). Clinical glove use: healthcare workers' actions and perceptions. J Hosp Infect.

